# Pulmonary Sarcoidosis Associated With Pleural Effusion: A Case Report

**DOI:** 10.1002/rcr2.70634

**Published:** 2026-05-27

**Authors:** Shpëtim Thaçi, Berat Krasniqi, Arben Redjepi, Mentor Sopjani

**Affiliations:** ^1^ Faculty of Medicine University of Prishtina Prishtina Kosovo; ^2^ Hospital Acibadem Sistina Skopje North Macedonia; ^3^ Department of Paramedicine University of Prishtina Prishtina Kosovo

**Keywords:** corticosteroids, EBUS‐TBNA, non‐caseating granulomas, pleural effusion, sarcoidosis

## Abstract

Sarcoidosis is a multisystem granulomatous disorder of unknown aetiology that predominantly affects the lungs and intrathoracic lymph nodes. Pleural involvement is uncommon, and sarcoidosis‐associated pleural effusion (SAPE) represents a rare clinical manifestation, reported in approximately 1% of cases. We describe a 24‐year‐old male presenting with bilateral pleural effusion and mediastinal lymphadenopathy. Comprehensive diagnostic evaluation revealed a lymphocyte‐predominant exudative pleural effusion with negative microbiological and cytological findings. Endobronchial ultrasound‐guided transbronchial needle aspiration (EBUS‐TBNA) demonstrated non‐caseating granulomas consistent with sarcoidosis. Elevated serum angiotensin‐converting enzyme (ACE), soluble interleukin‐2 receptor (sIL‐2R), and serum amyloid A (SAA) supported active disease. The patient achieved complete clinical, biochemical and radiological remission following systemic corticosteroid therapy. This case highlights the importance of a rigorous differential diagnostic approach and careful clinicoradiological–histopathological correlation in rare presentations of sarcoidosis.

## Graphical abstract

A rare case of pulmonary sarcoidosis presenting with bilateral pleural effusion is described in a young adult. Diagnosis was supported by characteristic radiological findings, lymphocyte‐predominant exudative pleural effusion and histological confirmation of non‐caseating granulomas via EBUS‐TBNA. Systemic corticosteroid therapy resulted in complete clinical and radiological resolution.

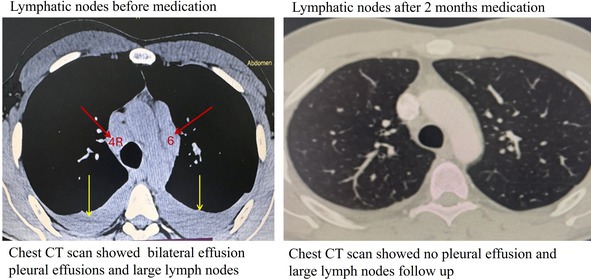

## 1 Introduction

Sarcoidosis is a systemic inflammatory disease characterized by the formation of non‐caseating granulomas in affected organs. Pulmonary involvement occurs in more than 90% of patients and commonly manifests as bilateral hilar lymphadenopathy with or without parenchymal infiltrates [1]. Although pleural manifestations—including pleural thickening, pleural nodules, pneumothorax and pleural effusion—have been reported, pleural effusion remains a distinctly uncommon feature [2, 3].

Sarcoidosis‐associated pleural effusion (SAPE) is typically exudative and lymphocyte‐predominant. Because tuberculosis, malignancy and connective tissue diseases are significantly more common causes of lymphocytic exudative effusions, SAPE is considered a diagnosis of exclusion [4, 5]. Histopathological confirmation of non‐caseating granulomas and systematic exclusion of alternative aetiologies are essential for accurate diagnosis.

We report a rare case of pulmonary sarcoidosis presenting with bilateral pleural effusion in a young adult, successfully treated with systemic corticosteroids.

## 2 Case Report

A 24‐year‐old previously healthy male presented with fever, progressive dyspnoea, non‐productive cough and intermittent abdominal discomfort. He denied weight loss, night sweats, haemoptysis or known tuberculosis exposure. Physical examination revealed reduced breath sounds bilaterally at the lung bases. No peripheral lymphadenopathy, cutaneous lesions or hepatosplenomegaly were detected.

**FIGURE 1:**
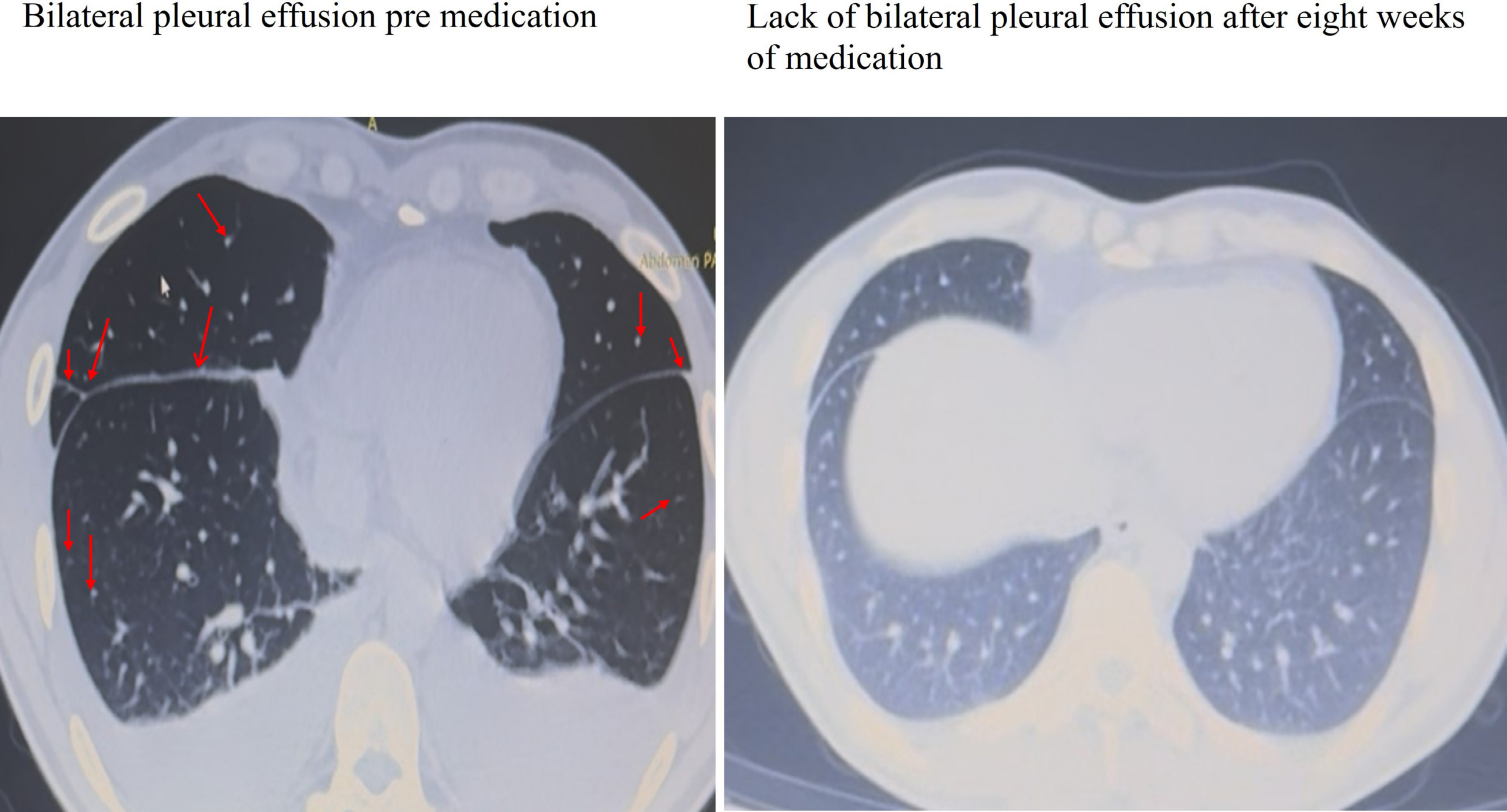
Baseline chest CT demonstrating bilateral pleural effusion and symmetrical mediastinal and hilar lymphadenopathy. Red arrows indicate multiple small nodules with a perilymphatic distribution.

**FIGURE 2:**
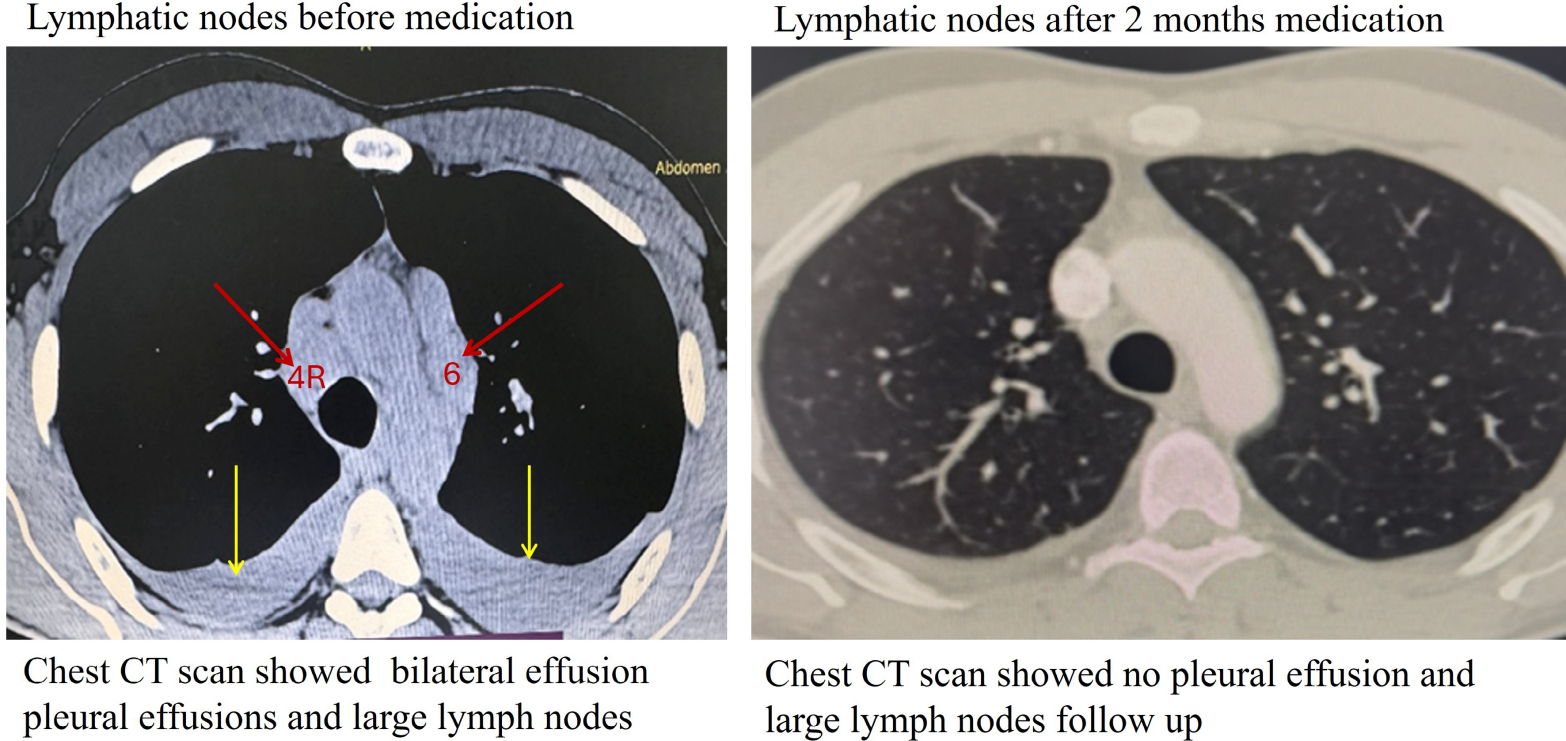
Follow‐up chest CT after 8 weeks of corticosteroid therapy showing complete resolution of pleural effusion and significant regression of lymphadenopathy. The yellow arrow indicates the previously identified pleural effusion. The red arrows indicate mediastinal lymph nodes, including station 4R (right lower paratracheal) and station 6 (para‐aortic) lymph nodes.

Contrast‐enhanced chest computed tomography (CT) demonstrated multiple small (3–4 mm), well‐defined nodules with a predominantly perilymphatic distribution, mainly involving the upper lobes. These nodules were distributed along bronchovascular bundles and subpleural regions, consistent with a typical perilymphatic pattern. In addition, symmetrical bilateral hilar and mediastinal lymphadenopathy (stations 4R, 4L, 5, 6, 7, 10R and 10L) was observed, consistent with typical radiological features of pulmonary sarcoidosis. Bilateral pleural effusion was also present. Follow‐up CT after eight weeks of corticosteroid therapy showed complete resolution of pleural effusion and marked regression of mediastinal and hilar lymphadenopathy with significant reduction in parenchymal nodules (Figures 1 and 2).

Routine haematological and biochemical investigations revealed no significant systemic inflammatory response. Erythrocyte sedimentation rate and C‐reactive protein were within normal limits, which is not uncommon in sarcoidosis and does not exclude active disease [1]. Mild elevations in hepatic transaminases (ALT and AST) were observed, possibly reflecting subclinical hepatic involvement, a recognized extrapulmonary manifestation of sarcoidosis [6]. Renal function, complete blood count and serum albumin levels were within normal ranges (Table 1).

**TABLE 1:** Laboratory findings.

Parameter	Patient value	Units	Referent range
ESR	2	mm/h	5–10
CRP	1.7	mg/L	1–6
WBC	4.9	× 10^3^/μl	4–10
Neutrophils	76.0	%	40–60
Lymphocytes	19.6	%	20–40
Eosinophils	3	%	0–6
Monocytes	4.4	%	2–8
RBC	5.89	× 10^6^/μl	3.8–5.2
MCV	81	fL	80–100
Haemoglobin	15.5	g/dL	11–16
Haematocrit	47.4	%	45–50
Platelets	250	× 10^3^/μl	150–400
Creatinine	99.8	μmol/L	58–110
Urea	4.53	mmol/L	3.2–7
ALT	53	U/L	< 40
AST	50	U/L	< 40
Total bilirubin	16.4	μmol/L	5.1–17
Direct bilirubin	4.2	μmol/L	1.7–5.1
Indirect bilirubin	3.4	μmol/L	3.4–12
D‐dimeri	1.55	mg/L	< 0.5
Albumin	43.4	g/ml	35–50
Total Protein g/L	67.6	g/ml	63–82
LDH	205	U/L	140–260
ADA	14.3	U/L	< 30
AFB stain/culture	Negative	—	—
PPD	Negative	—	—
IGRA	Negative	—	—

Tuberculosis screening with purified protein derivative (PPD) testing was negative, further supporting the exclusion of mycobacterial infection in conjunction with pleural fluid findings. Biomarkers associated with sarcoidosis activity were significantly elevated prior to treatment. Serum angiotensin‐converting enzyme (ACE) was increased (63 U/L), reflecting heightened granuloma burden, although ACE lacks sufficient specificity to be diagnostic on its own (Baughman et al., 2011). Soluble interleukin‐2 receptor (sIL‐2R), a marker of T‐cell activation and disease activity, was markedly elevated (80 mg/L), correlating with active granulomatous inflammation (Judson, 2015). Serum amyloid A (SAA), an acute‐phase reactant associated with chronic inflammatory conditions, was also substantially elevated. Following three months of systemic corticosteroid therapy, all three biomarkers normalized, paralleling the patient's clinical and radiological remission and further supporting the diagnosis of active sarcoidosis responsive to immunosuppressive therapy. Routine laboratory tests showed normal inflammatory markers. Liver transaminases were mildly elevated. Tuberculosis screening (PPD) and interferon‐gamma release assay (IGRA) were negative (Table 1).

Sarcoidosis‐associated biomarkers were elevated prior to treatment. After three months of corticosteroid therapy, values normalized (Table 2).

**TABLE 2:** Specific serum analysis for sarcoidosis prior and after three months of medication.

Parameter	Before treatment	After treatment	Units	Reference range
ACE	63	27	U/L	< 50
‐sIL‐2R	80	8.2	mg/L	< 10
SAA	2300	300	mg/L	150–600

Diagnostic thoracentesis was performed with simultaneous collection of paired serum and pleural fluid samples. Pleural fluid analysis met Light's criteria for an exudative effusion [4, 5], with a pleural fluid/serum protein ratio of 0.69 and a pleural fluid/serum LDH ratio of 0.88. The pleural fluid LDH also exceeded two‐thirds of the upper limit of normal serum LDH, further supporting exudate classification. The pleural fluid showed a protein concentration of 47 g/L, LDH of 180 U/L, a total leukocyte count of 4526/μL and a lymphocyte (count 4.5/μL) predominance of 70%. Cytological examination was negative for malignant cells, and microbiological studies, including acid‐fast bacilli staining, were negative. The ADA level was low (14.3 U/L), arguing against tuberculous pleuritis (Tables 3 and 4).

**TABLE 3:** Biochemical analysis of pleural fluid removed from the pleural effusion space.

Parameter	Value	Units
pH	7.42	—
Glucose	72	mg/dL
Protein	47	g/L
LDH	180	U/L
WBC	4.5	× 10^3^/μl
Lymphocytes	70	%
Cytology	Negative	—

**TABLE 4:** Pleural fluid analysis and Light’ criteria.

Parameter	Serum value	Pleural fluid value	Ratio/comparison	Meeting criteria
Protein (g/L)	67.6	47	0.69	Yes
LDH (U/L)	205	180	0.88	Yes
LDH pleural vs. 2/3 serum LDH	260 (ULN)	180	180 > 173.3 (2/3 × 260)	Yes

Analysis of pleural fluid and serum reveals Light's criteria indicative of exudative effusion. All three criteria are satisfied, confirming that the effusion is exudative (Table 4).

Importantly, cytological examination was negative for malignant cells, effectively reducing the likelihood of pleural carcinomatosis or lymphoma (Table 3). Furthermore, the adenosine deaminase (ADA) level was 14.3 U/L, well below the threshold commonly associated with tuberculous pleural effusion (> 40 U/L), thereby making tuberculosis unlikely [7]. Acid‐fast bacilli staining and mycobacterial cultures were also negative (Table 1). Collectively, the biochemical profile, lymphocytic predominance, low ADA activity, and negative microbiological and cytological findings supported a non‐infectious, non‐malignant granulomatous aetiology consistent with sarcoidosis.

**FIGURE 3:**
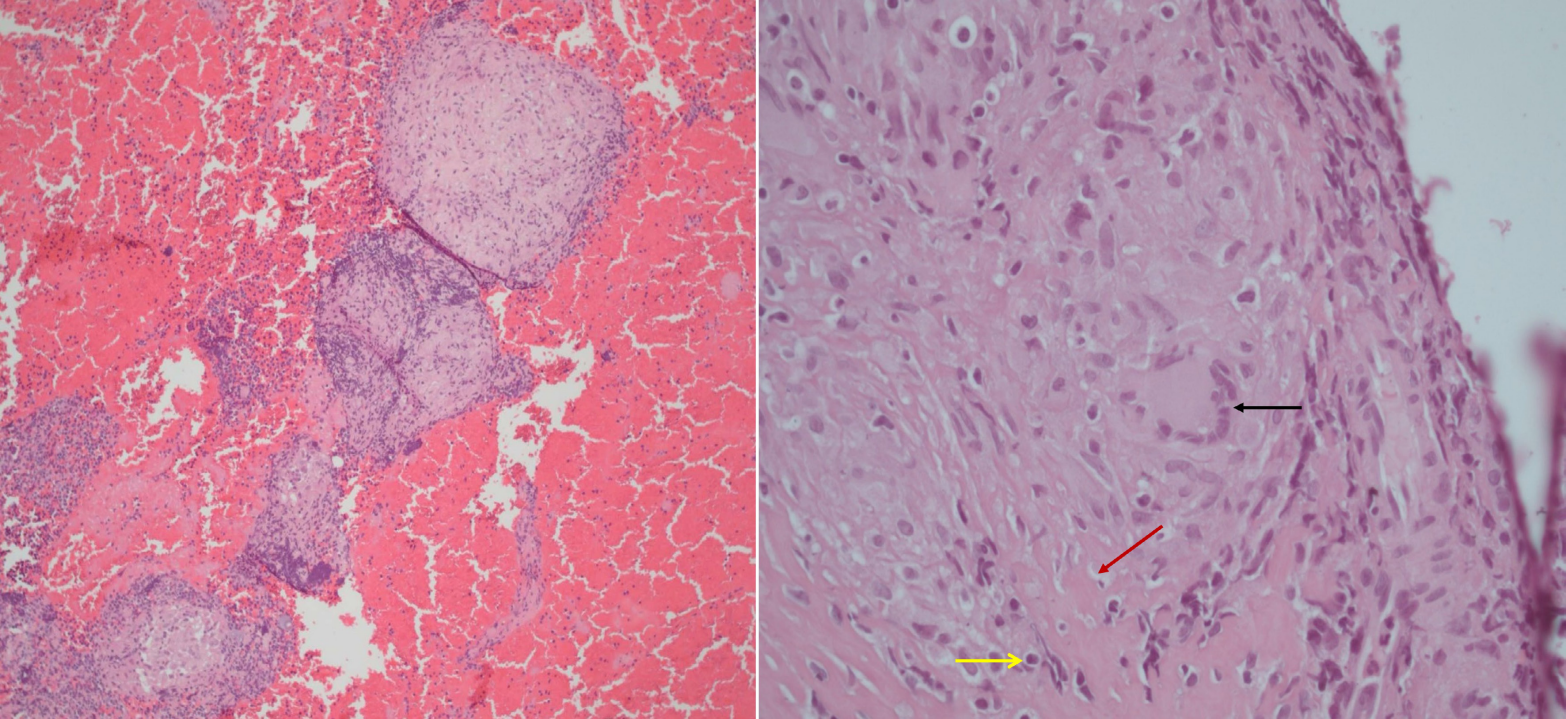
Histopathological examination of mediastinal lymph node tissue with coalesced non‐caseating granulomas. Low‐power view (haematoxylin and eosin stain, ×20) demonstrating multiple well‐formed, coalescent non‐caseating granulomas within the lymph node architecture (left image). High‐power view (haematoxylin and eosin stain, ×40) showing non‐necrotizing granulomatous inflammation composed of epithelioid histiocytes (red arrow), multinucleated giant cells (black arrow) and a peripheral rim (“corona”) of lymphocytes surrounding the granulomas and some individual histiocytes (yellow arrow) (right image).

Endobronchial ultrasound‐guided transbronchial needle aspiration (EBUS‐TBNA) of mediastinal lymph nodes revealed well‐formed, non‐caseating granulomas composed of epithelioid histiocytes and lymphocytes without caseous necrosis (Figure 3).

Collectively, clinical, radiological, laboratory and histopathological findings were consistent with pulmonary sarcoidosis and a presumptive diagnosis of sarcoidosis‐associated pleural effusion.

The patient was treated with systemic corticosteroids, initiating oral prednisone at 40 mg/day. Significant clinical improvement was observed within eight weeks, accompanied by complete radiological resolution of pleural effusion and regression of lymphadenopathy. The prednisone dose was tapered gradually every four weeks to a maintenance dose of 10 mg/day, with a total treatment duration of six months. No relapse was observed during follow‐up.

## 3 Discussion

Pleural effusion represents an uncommon manifestation of sarcoidosis, occurs in approximately 1% of patients, and may develop at any radiographic stage of the disease [3]. The underlying pathophysiology is not completely understood but is thought to involve direct granulomatous infiltration of the pleura, obstruction of pleural lymphatic drainage by mediastinal lymphadenopathy, and increased microvascular permeability driven by local cytokine‐mediated inflammation. These mechanisms collectively result in an inflammatory exudate within the pleural space.

Management of SAPE should be individualized. While spontaneous resolution may occur in asymptomatic or small effusions, systemic corticosteroid therapy is generally indicated for patients with symptomatic disease, significant pulmonary involvement, or a progressive clinical course [8]. In the present case, progressive dyspnoea, bilateral pleural effusion, extensive mediastinal lymphadenopathy and elevated biomarkers of disease activity indicated active systemic sarcoidosis and justified initiation of treatment. Prednisone was initiated at 40 mg/day, in line with recommended regimens for moderate pulmonary sarcoidosis (typically 20–40 mg/day), resulting in rapid clinical and radiological improvement [8]. A gradual tapering strategy was implemented over six months, guided by sustained clinical remission and radiological resolution, to minimize relapse risk while reducing corticosteroid‐related adverse effects [9].

SAPE is characteristically exudative and lymphocyte‐predominant, reflecting T‐cell–mediated immune activation within the pleural compartment. However, this profile overlaps substantially with tuberculous and malignant effusions, necessitating rigorous exclusion of alternative aetiologies. In particular, low ADA levels and negative microbiological studies substantially reduce the likelihood of tuberculous pleuritis, especially in endemic regions [7]. Cytological negativity and absence of radiographic features suggestive of malignancy further support a benign inflammatory cause. Definitive diagnosis of pleural involvement requires histopathological demonstration of non‐caseating granulomas; however, in clinical practice, SAPE is often diagnosed presumptively after exclusion of alternative aetiologies [1].

Systemic corticosteroids remain the cornerstone of therapy for symptomatic pulmonary sarcoidosis and pleural involvement. Reported cases of SAPE typically demonstrate rapid clinical and radiological resolution following corticosteroid initiation, with a generally favourable long‐term prognosis [1, 2, 3, 6]. This case emphasizes the value of a structured, evidence‐based diagnostic approach and illustrates the excellent therapeutic response achievable when SAPE is accurately identified and promptly treated.

A major limitation of this study case is the lack of a pleural biopsy, which prevents definitive histopathological confirmation of pleural involvement by sarcoidosis. As a result, the diagnosis of SAPE remains presumptive. Nevertheless, it is strongly supported by a combination of clinical, radiological and histopathological evidence: non‐caseating granulomas identified in mediastinal lymph nodes via EBUS‐TBNA, a lymphocyte‐predominant pleural effusion, low ADA levels, negative microbiological and cytological analyses, and a negative interferon‐gamma release assay (IGRA). Furthermore, the significant clinical and radiological improvement following corticosteroid therapy supports the diagnosis after alternative causes, particularly tuberculosis, have been excluded.

In conclusion, sarcoidosis‐associated pleural effusion is a rare but clinically significant medical condition. Accurate diagnosis requires thorough exclusion of infectious and malignant causes and integration of clinical, radiological and histopathological findings, although pleural involvement may remain presumptive in the absence of pleural biopsy.

## Author Contributions

**Shpëtim Thaçi:** conceptualization, data curation, formal analyses, investigation, writing – review and editing. **Berat Krasniqi:** conceptualization, data curation, formal analyses, writing – review and editing. **Arben Redjepi:** formal analyses, investigation, writing – review and editing. **Mentor Sopjani:** conceptualization, data curation, writing – original draft, writing – review and editing.

## Funding

The authors have nothing to report.

## Consent

The authors declare that written informed consent was obtained for the publication of this manuscript and accompanying images using the consent form provided by the journal.

## Conflicts of Interest

The authors declare no conflicts of interest.

## Data Availability

The data that support the findings of this study are available from the corresponding author upon reasonable request.
